# Outcomes of a Novel Percutaneous Achilles Tendon Repair Technique: A Retrospective Case Series

**DOI:** 10.7759/cureus.90546

**Published:** 2025-08-20

**Authors:** Meraj Akhtar, Uday Mahajan, Kar H Teoh, Paul Lee

**Affiliations:** 1 Trauma and Orthopaedics, Lincoln County Hospital, Lincoln, GBR; 2 Trauma and Orthopaedics, Queen Elizabeth Hospital Birmingham, Birmingham, GBR; 3 Trauma and Orthopaedics, Princess Alexandria Hospital Trust, Harlow, GBR; 4 Orthopaedics, Lincoln County Hospital, Lincoln, GBR

**Keywords:** achilles tendon rupture, disposable instruments, functional outcomes, minimally invasive surgery, percutaneous repair

## Abstract

Background: The management of acute mid-substance Achilles tendon ruptures has shifted towards minimally invasive techniques to reduce soft tissue complications. However, many percutaneous repair methods require expensive, dedicated instrumentation or reusable devices with reprocessing needs. This study describes a novel percutaneous Achilles tendon repair technique using readily available disposable standard instruments (Achilles-tendon Soft Tissue Augmentation Regenerative Repair (A-STARR)) and evaluates its clinical outcomes.

Methods: A retrospective case series was conducted involving 21 patients (18 male, 3 female) with confirmed acute mid-substance Achilles tendon ruptures. All underwent percutaneous repair using the A-STARR technique between July 2017 and May 2018. Patients were followed up for a minimum of 12 months (range: 12-25 months). Primary outcomes included complication rates, time to return to work, return to sport, and the Achilles Tendon Total Rupture Score (ATRS) at one year.

Results: No wound infections, re-ruptures, or sural nerve injuries were observed. The mean time to return to work was 40 days (range: 30-92 days). 18 patients (85.7%) returned to their pre-injury sport at a mean of 6.5 months (range: 4-12 months). The mean ATRS at one-year follow-up was 83.1 (range: 70-95).

Conclusion: The A-STARR percutaneous Achilles tendon repair using disposable standard instruments was safe and effective in this series, with no complications and good functional recovery. It may provide a practical and cost-efficient alternative for treating acute mid-substance ruptures, particularly where specialized equipment is unavailable.

## Introduction

Acute Achilles tendon ruptures are common injuries, with an incidence ranging from 7 to 40 per 100,000 person-years [1]. The rising participation of older adults in recreational and competitive sports, combined with the increasing prevalence of metabolic and chronic diseases, has contributed to a growing incidence of these injuries [1,2]. Ruptures most frequently occur in the mid-substance region, approximately 2-6 cm proximal to the calcaneal insertion, an area with relatively poor vascularity [2]. They are more commonly seen in men aged 30-45 years, particularly among recreational athletes.

Accurate and timely diagnosis is essential, as up to 20-25% of Achilles tendon ruptures are initially missed [2]. Delayed treatment can lead to tendon discontinuity or lengthening, resulting in functional deficits that may require more complex reconstructive procedures.

The optimal management of acute Achilles tendon ruptures remains controversial. Historically, nonoperative treatment with prolonged immobilization was associated with higher re-rupture rates compared to surgical repair, whereas surgical intervention carried higher risks of wound complications and sural nerve injuries [3,4]. The introduction of functional nonoperative rehabilitation with early weight bearing has shifted management paradigms, with some centers reporting excellent outcomes using conservative treatment protocols [5,6].

Among surgical options, minimally invasive percutaneous repair techniques have gained popularity due to their reduced risk of wound complications compared to open repairs [7,8]. Techniques such as the Dresden method and commercial systems like Achillon and Percutaneous Achilles Repair System (PARS) have shown favorable outcomes, but they often require dedicated, expensive instrumentation or reusable devices requiring reprocessing [9,10]. With the increasing emphasis on cost-effective healthcare models, reducing sterilization needs and operating room inefficiencies has become a priority [11]. In light of these limitations, we developed Achilles-tendon Soft Tissue Augmentation Regenerative Repair (A-STARR) technique to address these shortcomings by offering a cost-effective, easy-to-use method that can be used as a single-use disposable. By simplifying the repair process and reducing dependence on costly specialized tools, this approach aims to make minimally invasive Achilles tendon repair more accessible without compromising clinical outcomes.

This study describes a novel percutaneous A-STARR technique using readily available, disposable standard instruments and evaluates its clinical outcomes. We hypothesized that this approach would provide safe, effective tendon repair with minimal complications, while offering operational advantages by eliminating the need for expensive or reusable specialized equipment.

## Materials and methods

Study design and patient cohort

This was a retrospective case series of 21 patients (18 male, 3 female) with confirmed acute mid-substance Achilles tendon ruptures who underwent percutaneous repair using the A-STARR technique between July 2017 and May 2018. The mean age at injury was 38.2 years (range: 22-51 years, SD: 7.32). All patients had a minimum follow-up of 12 months, with follow-up duration ranging from 12 to 25 months.

Inclusion and exclusion criteria

Patients were included if they had an acute mid-substance Achilles tendon rupture diagnosed clinically within six weeks of injury. Surgical repair was indicated for high-demand patients, including elite athletes and young active individuals with sporting aspirations, as well as for ruptures demonstrating a tendon gap exceeding 1 cm on plantarflexion, assessed clinically or with ultrasound. Exclusion criteria included patients with diabetes mellitus, current smokers, those with chronic steroid use, peripheral vascular disease, local skin infections, or ruptures associated with open wounds, as these were unsuitable for the percutaneous approach described.

Outcome measures

Primary outcomes assessed were complication rates, including wound infection, re-rupture, and sural nerve injury. Secondary outcomes included time to return to work, return to sport (defined as return to pre-injury activity level and time taken), and the Achilles Tendon Total Rupture Score (ATRS) at one-year follow-up. Data were collected retrospectively from medical records and clinic follow-up assessments. The ATRS is a validated, patient-reported outcome measure specifically designed for individuals with acute Achilles tendon rupture [12]. It comprises 10 items, with responses recorded on an 10-point Likert scale ranging from 0 to 10. A score of 0 indicates major symptoms or limitations, while a score of 10 denotes no symptoms or limitations. The ATRS assesses two primary constructs: symptoms and physical activity, with five questions dedicated to each domain. A higher ATRS score reflects better functional outcomes. The ATRS has demonstrated high reliability, validity, and responsiveness, making it an effective tool for assessing recovery and treatment efficacy in patients with total Achilles tendon ruptures. We obtained formal permission to use the ATRS in this study from the original copyright holders, and the instrument was used under license number 6074310634836.

Preoperative planning

All patients underwent a thorough clinical evaluation prior to surgery. History typically revealed a sudden painful blow to the posterior ankle, often accompanied by an audible pop or snap, with immediate difficulty weight bearing. Injuries usually resulted from an indirect mechanism involving forced dorsiflexion of a plantarflexed foot, and patients reported weakness during push-off in gait.

Risk factors assessed included age, prior Achilles tendinopathy or surgery, systemic or local corticosteroid use, quinolone antibiotic use, and relevant comorbidities. Occupational demands, lifestyle, and sporting aspirations were documented to guide treatment decisions.

On examination, patients were assessed prone with feet hanging over the examination couch. Findings included bruising and swelling over the tendon, a palpable tendon gap, reduced resting plantarflexion tension, and decreased plantarflexion power. The Thompson (Simmonds) test and Matles test were performed. The presence of Simmonds triad (gap, loss of resting tension, positive calf squeeze test) was used to confirm diagnosis, with reported 100% sensitivity. Neurovascular assessment, particularly of the sural nerve, was performed in all patients.

Imaging was used selectively as an adjunct to clinical assessment. Ultrasound was employed when needed to confirm rupture localization and assess tendon end apposition, aiding operative planning. MRI was not routinely performed, as it was deemed to provide limited additional diagnostic value and risk delaying treatment. Informed consent was taken from all patients prior to surgery explaining all risk and benefit.

Surgical technique

All procedures were performed under local anesthesia by orthopedic consultant with sub-speciality interest in foot and ankle surgeries. The anesthetic solution consisted of 10 ml of 2% lidocaine, 1 ml of 1:1000 adrenaline (1 mg), and 10 ml of 0.5% chirocaine, diluted in 100 ml normal saline. This was infiltrated around the operative site using multiple syringes to achieve adequate field block.

Patients were positioned prone, with feet hanging off the distal edge of the operating table, and all pressure points were padded. A tourniquet was not used. The operative leg was prepped to knee level with chlorhexidine, and IV cefuroxime was administered preoperatively.

The equipment required included a disposable needle passer, 3 mm Vicryl tape, and a No. 15 blade scalpel. The rupture site was confirmed clinically and with intraoperative ultrasound marking. Four small longitudinal stab incisions were made, two proximal and two distal to the rupture site, approximately 6 cm from the palpable gap. Care was taken to avoid injury to the sural nerve, and blunt dissection with a hemostat was used when necessary to facilitate suture passage.

**Figure 1:**
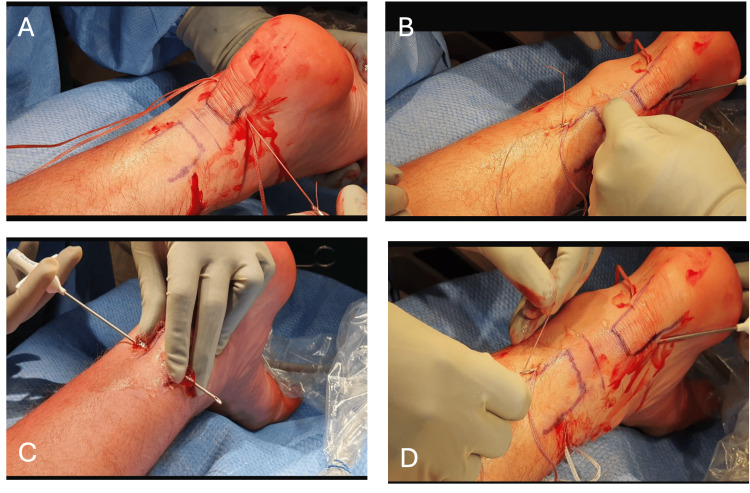
Intraoperative sequence of the A-STARR percutaneous Achilles tendon repair technique Panels A–D (clinical images) show corresponding intraoperative steps: (A) Four stab incisions created; (B) Suture passed on the distal tendon stump using a suture passer from medial to lateral; (C) Suture passed proximally through the tendon; (D) Suture tied proximally to secure the repair. A-STARR: Achilles-tendon Soft Tissue Augmentation Regenerative Repair

Using the needle passer, the Vicryl tape was passed from medial to lateral distally, approximately 1-2 cm proximal to the Achilles insertion on the calcaneus. The tape was then retrieved proximally through the rupture site with a strong bite through the tendon substance to ensure secure purchase. A secure knot was tied proximally and buried under the skin to minimize irritation. Tendon apposition was confirmed by tensioning the suture, comparing the resting plantarflexion angle with the contralateral side, and palpating the repair site. Skin incisions were closed with steri-strips and skin glue. Sterile dressings were applied, and the ankle was immobilized in 20° plantarflexion using a below-knee backslab (see Figure 1).

Postoperative care

At two weeks post operation, the backslab was removed, and patients were transitioned into a Vacoped boot locked at 30° with full weight bearing. The boot remained locked at 30° until week five. Between weeks five and seven, the boot was adjusted from 30° to 15°, and yellow theraband exercises for gastrocnemius and soleus strengthening were initiated. Between weeks seven and nine, the boot was unlocked from 0° to 30° with full weight bearing using a flat wedge. By weeks nine to ten, the boot was fully unlocked with continued full weight bearing. After 10 weeks, the boot was removed entirely but was advised to be worn in vulnerable environments for an additional six weeks.

All patients received deep vein thrombosis (DVT) prophylaxis with Clexane for six weeks. Functional rehabilitation commenced two weeks post operation, focusing initially on gradual range of motion exercises, then progressing to strengthening, proprioception, and plyometric training according to standard rehabilitation protocols.

## Results

21 patients (18 male and 3 female) with acute mid-substance Achilles tendon ruptures underwent percutaneous repair using the A-STARR technique between July 2017 and May 2018. The mean age at injury was 38.2 years (range: 22-51 years, SD: 7.32). All patients completed a minimum of 12 months of follow-up, with the range extending up to 25 months.

There were no recorded complications related to the procedure. Specifically, there were no cases of wound infection, re-rupture, or sural nerve injury observed in this series.

The mean time to return to work was 40 days, with a range of 30 to 92 days. Regarding sporting activity, 18 patients (85.7%) were able to return to their pre-injury sport at a mean of 6.5 months (range: 4-12 months). At one-year follow-up, the mean ATRS was 83.1 (range: 70-95), indicating good to excellent functional outcomes across the cohort. The summary is mentioned in Table 1.

**Table 1:** Summary of results ATRS: Achilles Tendon Total Rupture Score

Outcome Measure	Result
Number of patients	21 (18 male, 3 female)
Age (years)	Mean 38.2 (range: 22–51, SD: 7.32)
Follow-up duration (months)	Minimum 12 (range: 12–25)
Wound infection	0
Re-rupture	0
Sural nerve injury	0
Mean time to return to work (days)	40 (range: 30–92)
Return to sport	85.7% at mean 6.5 months (range: 4–12)
Mean ATRS at 1 year	83.1 (range: 70–95)

## Discussion

Acute mid-substance Achilles tendon ruptures remain a common and challenging injury, and the optimal repair method continues to be debated. This study describes the outcomes of a novel percutaneous Achilles tendon repair technique using disposable standard instruments (A-STARR) and demonstrates its safety and effectiveness in treating acute mid-substance Achilles tendon ruptures.

The primary findings were favorable, with no recorded complications, including wound infections, re-rupture, or sural nerve injury. Patients achieved a mean ATRS of 83.1 at one year, indicating good to excellent functional outcomes, and 85.7% returned to their previous sport at a mean of 6.5 months. The mean time to return to work was 40 days, demonstrating early functional recovery.

These results compare favorably to published outcomes of existing percutaneous repair techniques. The Dresden technique, for example, reported re-rupture rates of 2-3% with mean return to work at 56 days and return to sport at approximately 19 weeks [9,10]. Our cohort had no re-ruptures, an earlier mean return to work (40 days), and return to sport by an average of 6.5 months (~26 weeks), aligning well with existing literature [11,13].

Recent studies have highlighted that surgical repair reduces re-rupture risk compared to conservative management when early functional rehabilitation is not employed [14-19]. Soroceanu et al. found an absolute re-rupture risk reduction of 8.8% with surgery if early range of motion was not implemented, although surgical treatment carried a 15.8% higher risk of complications such as infection or nerve injury [14]. Our findings suggest that minimally invasive percutaneous repair using disposable instruments can mitigate these surgical risks, as evidenced by the absence of wound complications or nerve injuries in our series.

Functional outcomes following surgical versus conservative treatment remain debated. Nilsson-Helander et al. reported no significant differences in ATRS at 6 and 12 months between surgical and non-surgical groups when early mobilization was used, though surgical patients showed better muscle function at six months [20]. Willits et al. found no clinically important differences in strength or range of motion at two years but did note improved plantarflexion strength in surgically treated patients [15]. Our cohort achieved similar ATRS results to those reported in these trials, supporting the effectiveness of surgical repair combined with functional rehabilitation [9,10].

A key advantage of our technique lies in its use of readily available disposable instruments, avoiding the need for expensive dedicated kits such as the Achillon or PARS systems. Studies comparing Achillon and open repairs have shown significantly reduced complication rates with percutaneous techniques, without differences in re-rupture rates or return to sport [7-9]. The A-STARR technique provides similar benefits while enhancing operating room efficiency, reducing reprocessing costs, and maintaining patient safety.

This study has limitations. It is a retrospective, case series with a relatively small sample size (21 patients) and no control group, which limits the ability to establish causality or directly compare A-STARR with other techniques. The absence of a control group also introduces potential selection bias. Future studies with larger cohorts, long-term outcomes, and comparative designs are needed to further validate these results. Overall, our findings support A-STARR as a safe and effective repair option that can be implemented without the need for specialized equipment.

## Conclusions

This study found that the A-STARR percutaneous Achilles tendon repair technique using disposable standard instruments demonstrated good to excellent clinical outcomes with no recorded complications. The method is reproducible, cost-efficient, and avoids the need for dedicated repair systems, making it a viable option in diverse surgical environments. Larger comparative studies are needed to further evaluate its long-term effectiveness.
